# Miscarriage history is not associated with embryonic aneuploidy in women under 38 years of age with recurrent pregnancy loss

**DOI:** 10.3389/fendo.2026.1912686

**Published:** 2026-07-23

**Authors:** Sui-Bing Miao, Jing-Chuan Yuan, Shu-Hong Pan, Yun-Ying Li, Xiao-Hua Wu, Li-Xia Wu

**Affiliations:** 1 Institute of Reproductive Medicine of Shijiazhuang, The Fourth Hospital of Shijiazhuang, Shijiazhuang, China; 2 IVF Laboratory, Centre of Reproductive Medicine, The Fourth Hospital of Shijiazhuang, Shijiazhuang, China; 3 Department of Gynecology, Shijiazhuang People’s Hospital, Shijiazhuang, China; 4 Department of Obstetrics, The Fourth Hospital of Shijiazhuang, Shijiazhuang, China

**Keywords:** embryonic aneuploidy, maternal age, number of miscarriages, PGT−A, recurrent pregnancy loss

## Abstract

**Purpose:**

This study aimed to determine whether embryonic aneuploidy rates differ between patients with and without recurrent pregnancy loss (RPL).

**Methods:**

This retrospective cohort study included 103 patients with RPL undergoing 128 preimplantation genetic testing for aneuploidy (PGT-A) cycles and 83 controls undergoing 125 preimplantation genetic testing for monogenic defects (PGT−M) cycles, all aged <38 years. Baseline characteristics, ovarian stimulation parameters, and embryologic data were compared. Miscarriage-number subgroup and age-stratified analyses were performed. A modified Poisson regression model evaluated independent effects of the number of prior miscarriages and age on aneuploidy risk.

**Results:**

The RPL group had a significantly higher mean age than that of the control group (33.0 ± 2.9 years vs. 31.2 ± 3.7 years, *P* < 0.001), as well as a lower antral follicle count, oocyte yield, and number of metaphase II oocytes. No significant difference in embryonic aneuploidy rates was observed between groups, in the overall analysis and after stratification by age. A subgroup analysis according to the number of prior miscarriages also revealed no significant difference between groups. A multivariable analysis revealed that the number of prior miscarriages was not associated with aneuploidy risk (adjusted RR: 0.999, 95% CI 0.984–1.015, *P* = 0.923), and maternal age exhibited a trend toward increased risk of embryonic aneuploidy but this relationship did not reach statistical significance (adjusted RR: 1.007, 95% CI 1.001–1.013, *P* = 0.022).

**Conclusion:**

Among patients with RPL aged <38 years, miscarriage history is not associated with embryonic aneuploidy, suggesting that embryonic chromosomal aneuploidy is not the primary determinant of RPL in this population, warranting cautious use of PGT-A alongside comprehensive etiological evaluation.

## Introduction

Recurrent pregnancy loss (RPL), typically defined as two or more consecutive pregnancy losses before 20 weeks of gestation, affects approximately 1%–5% of pregnant individuals (1). The etiology of RPL is multifaceted, encompassing chromosomal abnormalities, uterine anatomical anomalies, endocrine disorders, immunological factors, thrombophilic conditions, and environmental influences. Despite extensive evaluation, approximately 50% of cases remain unexplained (2), posing significant clinical challenges and psychological burdens for affected couples.

Embryonic chromosomal abnormalities, particularly aneuploidy, are a well-established cause of early pregnancy loss. It is estimated that more than half of spontaneous miscarriages in the first trimester are attributable to embryonic aneuploidy (3), with the proportion increasing with advancing maternal age (4). Given the high prevalence of chromosomal aberrations in miscarried products of conception, preimplantation genetic testing for aneuploidy (PGT-A), a technique that screens embryos for numerical chromosomal abnormalities, is increasingly utilized in patients with RPL to select euploid embryos for transfer, theoretically reducing the risk of subsequent miscarriage (5).

However, the role of PGT-A in improving clinical outcomes for patients with RPL remains a subject of debate. While some studies have reported reduced miscarriage rates following euploid embryo transfer in this population, others have questioned the cost-effectiveness and overall benefit, particularly given that RPL may be driven by mechanisms beyond embryonic aneuploidy (6, 7). Some investigators have suggested that RPL may be associated with a higher incidence of aneuploidy in subsequent pregnancies, whereas others have found no such association (8–10). Moreover, the relationship between the number of prior miscarriages and the risk of embryonic aneuploidy is not well defined.

This retrospective study compared the embryonic aneuploidy rates between patients with or without RPL using aneuploidy screening data from preimplantation genetic testing. The study results provide insight into the association between chromosomal aneuploidy and RPL, as well as the applicability of PGT-A in RPL.

## Materials and methods

### Study design and population

This was a retrospective cohort study conducted at the Fourth Hospital of Shijiazhuang between February 2019 and August 2025. Institutional Review Board approval was obtained for this study.

Patients who underwent PGT for recurrent pregnancy loss or for monogenic disorders were included and subsequently assigned to two groups based on their indication: the RPL group and the control group (monogenic disorders). The RPL group consisted of 103 patients aged <38 years with a history of 2–6 miscarriages and underwent 128 PGT−A cycles; the control group consisted of 83 couples aged <38 years with no or sporadic miscarriage and underwent 125 preimplantation genetic testing for monogenic defects (PGT−M) cycles.

Exclusion criteria for both groups were: (1) uterine anatomical abnormalities; (2) parental chromosomal abnormalities; (3) endocrine disorders; (4) thrombophilic disorders; (5) antiphospholipid syndrome or other autoimmune abnormalities; (6) genital tract infections; (7) male factor infertility requiring testicular sperm extraction.

### Treatment procedures

The experimental procedures were performed as previously described (11). The gonadotropin-releasing hormone agonist (GnRH−a) or antagonist (GnRH−ant) protocol was selected according to individual patient characteristics. The human chorionic gonadotropin was administered when there were two or more follicles with maximal diameters of 18 mm or greater in the ovaries. Transvaginal ultrasound-guided oocyte retrieval under deep conscious sedation was performed 36 h later.

Oocytes were fertilized via conventional intracytoplasmic sperm injection (ICSI) procedures. Embryos were cultured to the blastocyst stage in sequential media under standard conditions (37°C, 6% CO_2_ and 5% O_2_).

Trophectoderm biopsy was performed via a laser pulse on days 5–6 of development, and 4–6 cells were carefully aspirated and biopsied using a laser system for PGT. Biopsied samples were transferred into RNase- and DNase-free PCR tubes containing 5 μL of cell lysis buffer. Blastocysts were vitrified after biopsy. After cell lysis, the samples were amplified via multiple annealing and looping-based amplification cycles. Comprehensive chromosomal screening was carried out using next-generation sequencing (NGS) on the Illumina HiSeq 2500 platform (Illumina, Inc.). The sequencing yielded copy number variation (CNV) results with approximately 4 Mb resolution. Embryos were classified as aneuploid if any numerical chromosomal abnormality was detected and as euploid if all 23 pairs of chromosomes were normal. Mosaic embryos were classified according to the laboratory’s standard criteria. Both euploid and mosaic embryos were collectively defined as non-aneuploid. The primary outcome was aneuploidy rate, expressed as number of aneuploid embryos divided by the number of blastocysts tested.

### Statistical analysis

Continuous data are presented as mean and standard deviation, and categorical variables are presented as absolute and percentage frequency. Student’s t-test or non-parametric test was used to examine the differences in continuous variables between groups, whereas Chi-square test was used to assess categorical variables. A modified Poisson regression model with robust error variance was used to evaluate the association among maternal age, the number of prior miscarriages, and embryonic aneuploidy rate. *P* < 0.01 was considered statistically significant. All statistical analyses were performed using SPSS 26.0.

## Results

In total, 128 PGT cycles in the RPL group and 125 PGT cycles in the control group were analyzed. Baseline characteristics of the two groups are summarized in **Table 1**. The mean age of women in the RPL group was 1.8 years older than that in the control group (*P* < 0.001), and the body mass index was comparable between the groups. The antral follicle count (AFC) was significantly lower in the RPL group than in the control group (*P* < 0.001). Among basal hormone levels, basal estradiol (E_2_) levels were higher in the RPL group than in the control group, while the baseline levels of follicle-stimulating hormone (FSH), luteinizing hormone (LH), and progesterone (P) were similar between the two groups.

**Table 1:** Baseline characteristics.

Variable	Control N = 125 mean ± SD	RPL N = 128 mean ± SD	*P*-value
Maternal age (years)	31.2 ± 3.7	33.0 ± 2.9	< 0.001
BMI (kg/m^2^)	22.7 ± 2.9	23.1 ± 3.0	0.163
AFC	19.8 ± 7.9	16.1 ± 9.7	< 0.001
Basal FSH (mIU/ml)	5.2 ± 1.3	5.5 ± 1.5	0.442
Basal LH (mIU/ml)	3.7 ± 1.8	3.7 ± 2.1	0.538
Basal E_2_ (pg/ml)	34.9 ± 19.9	38.6 ± 18.1	0.031
Basal P (ng/ml)	0.31 ± 0.19	0.29 ± 0.16	0.788

BMI, body mass index; AFC, antral follicle count; FSH, follicle-stimulating hormone; LH, luteinizing hormone; E_2_ estradiol; P, progesterone; SD, standard deviation.

Controlled ovarian hyperstimulation (COH) parameters and embryologic data are presented in **Table 2**. No significant difference was observed in the choice of ovarian stimulation protocol between the two groups, with the GnRH-antagonist protocol being the most commonly used. The duration of gonadotropin stimulation and cumulative gonadotropin dose were also comparable between the two groups. On the day of trigger, serum E_2_ levels were significantly higher in the control group than in the RPL group (*P* < 0.001), while no significant differences were observed in LH or P levels between the two groups. The numbers of aspirated oocytes, metaphase II (MII) oocytes, and two pronuclei (2PN) zygotes were significantly higher in the control than in the RPL group (*P* < 0.001, *P* < 0.001, and *P* = 0.003, respectively).

**Table 2:** COH parameters and embryologic data.

Variable	Control N = 125 mean ± SD or N (%)	RPL N = 128 mean ± SD or N (%)	*P*-value
Ovarian stimulation protocol			0.122
Long-acting GnRH-a protocol	14 (11.2%)	8 (6.3%)	
GnRH-ant protocol	107 (85.6%)	110 (85.9%)	
Other	4 (3.2%)	10 (7.8%)	
Duration of gonadotropin treatment	10.0 ± 1.5	9.7 ± 1.6	0.325
Cumulative gonadotropin dose (IU)	2575.8 ± 754.4	2505.7 ± 862.9	0.766
LH on day of trigger (mIU/ml)	1.9 ± 1.4	2.1 ± 1.3	0.174
E_2_ on day of trigger (pg/ml)	3523.4 ± 1325.7	2708.3 ± 1560.1	< 0.001
P on day of trigger (ng/ml)	1.2 ± 0.6	1.1 ± 0.7	0.057
Oocytes retrieved	11.9 ± 6.1	9.6 ± 6.3	< 0.001
MII oocytes	11.9 ± 6.1	9.6 ± 6.3	< 0.001
2PN zygotes	10.4 ± 5.8	8.5 ± 5.7	0.003

GnRH-a, gonadotropin-releasing hormone agonist; GnRH-ant, gonadotropin-releasing hormone antagonist; LH, luteinizing hormone; E_2_ estradiol; P, progesterone; MII, metaphase II; 2PN, two pronuclei; SD, standard deviation.

PGT analyses revealed no significant difference in the rates of aneuploid embryos between the two groups. Given the significant age difference between the groups and the well-established role of maternal age in embryonic aneuploidy, we performed age-stratified analyses, which also showed no significant differences within any age subgroup. The age−stratified analysis (**Table 3**) indicated that maternal age did not significantly affect the comparison of embryonic aneuploidy rates between the two groups.

**Table 3:** Distribution of aneuploid embryos between the control and RPL groups stratified by maternal age.

Variable	Aneuploid N (%)	Non-aneuploid N (%)	*P*-value
Maternal age 22–37 years			0.812
Control	151 (26.0%)	430 (74.0%)	
RPL	135 (26.7%)	372 (73.4%)	
Maternal age 22–27 years			0.366
Control	37 (25.2%)	110 (74.8%)	
RPL	4 (16.7%)	20 (83.3%)	
Maternal age 28–32 years			0.405
Control	52 (24.0%)	165 (76.0%)	
RPL	48 (20.7%)	184 (79.3%)	
Maternal age 33–37 years			0.294
Control	62 (28.6%)	155 (71.4%)	
RPL	83 (33.1%)	168 (66.9%)	

To further investigate whether the number of prior miscarriages is associated with embryonic aneuploidy rate, patients in the RPL group were subdivided according to the number of prior miscarriages (2 to 6 miscarriages) and compared with the control group (0 to 1 miscarriage) (**Table 4**). The results showed no significant differences in the distribution of aneuploid and non-aneuploid embryos between the control group and any of the RPL subgroups. No clear trend in embryonic aneuploidy rates was observed with increasing numbers of miscarriages. Notably, the sample sizes for the subgroups with five or more miscarriages were limited, and the findings for these subgroups should be interpreted with caution.

**Table 4:** Distribution of embryonic aneuploidy rates between the control group and RPL subgroups stratified by the number of prior miscarriages.

Variable	Aneuploid N (%)	Non-aneuploid N (%)	*P*-value
Control (0–1)	151 (26.0%)	430 (74.0%)	0.258
RPL (2)	72 (26.2%)	203 (73.8%)	
RPL (3)	37 (23.6%)	120 (76.4%)	
RPL (4)	23 (39.0%)	36 (61.0%)	
RPL (5)	2 (15.4%)	11 (84.6%)	
RPL (6)	1 (33.3%)	2 (66.7%)	

To evaluate the associations of the number of prior miscarriages and maternal age with embryonic aneuploidy rate, a modified Poisson regression model was used, and the results are presented in **Table 5**. After adjustment, the number of prior miscarriages showed no association with embryonic aneuploidy (adjusted RR = 0.999, 95% CI 0.984–1.015, *P* = 0.923). Maternal age was not significantly associated with embryonic aneuploidy at the predefined significance level of *P* < 0.01, although a trend was observed (adjusted RR = 1.007, 95% CI 1.001–1.013, *P* = 0.022). Therefore, the observed recurrence of pregnancy loss in these patients cannot be attributed to an increased rate of embryonic aneuploidy based on the current data.

**Table 5:** Modified Poisson regression analysis of the associations of maternal age and the number of prior miscarriages with the risk of embryonic aneuploidy.

Variable	*P*	Adjusted RR	95% CI
Maternal age	0.022	1.007	1.001–1.013
Number of prior miscarriages	0.923	0.999	0.984–1.015

## Discussion

In this study, among women aged <38 years, no significant difference was found in the rates of aneuploidy between the RPL and control groups, with or without adjusting for maternal age. Moreover, a stratified analysis by the number of prior miscarriages revealed no significant difference between groups. A multivariate regression analysis revealed that the number of prior miscarriages did not increase the risk, and maternal age was not a statistically significant independent risk factor for embryonic aneuploidy at *P* < 0.01, though a trend was present (*P* = 0.022). Taken together, the probability of numerical chromosomal aberrations in embryos from patients with RPLwas not different from that in controls without a history of RPL.

The key finding of the present study were that the rates of aneuploidy did not differ significantly between RPL group and control groupsand there was no association between the number of prior miscarriagesand embryonic aneuploidy. Based on these findings, it is reasonable to infer that the probability of miscarriage resulting from embryonic aneuploidy should be similar in the RPL and control groups. Hence, the increased number of miscarriages experienced by patients with RPL is more likely to be attributable to factors other than aneuploid embryos, which may be either stochastic or deterministic. These observations support the notion that in patients with multiple miscarriages, subsequent pregnancy losses may be attributable to non-chromosomal etiologies (12). Our findings further indicate that embryonic chromosomal aneuploidy alone cannot be regarded as the primary determinant of RPL. Instead, the occurrence of RPL appears to be a consequence of the combined effects of embryonic aneuploidy and multiple maternal factors, such as immune dysregulation, endocrine disorders, and thrombophilic predisposition (1). The relative contributions of these non-chromosomal factors to RPL warrants further investigation and confirmation through research focusing on clinical IVF outcomes. These findings have important clinical implications. They reinforce the current guideline recommendations for comprehensive etiological evaluation in patients with recurrent miscarriage. Relying solely on PGT-A to select euploid embryos may not fully address pregnancy failure in patients with RPL, as a subset of miscarriages may be unrelated to embryonic aneuploidy. This is consistent with the findings of Liu et al., who observed that even after euploid embryo transfer, the clinical miscarriage rates remained higher in young patients with idiopathic RPL than in controls (26.1% vs. 3.1%), underscoring the important role of maternal factors beyond embryonic aneuploidy (13).

In the present study, there was a trend for maternal age to increase the risk of embryonic aneuploidy in women younger than 38 years (*P* = 0.022), although this trend did not reach statistical significance at the predefined threshold of *P* < 0.01. This observation aligns with a growing body of evidence suggesting that the relationship between maternal age and aneuploidy risk is not linear but rather follows a biphasic or threshold pattern, with a marked acceleration after the mid 30s. The increasing risk of embryonic aneuploidy with advancing maternal age is well-established. However, this increase is not uniform across all age groups. A landmark study by Pendina et al. analyzing 7,118 miscarriages reported that the overall incidence of chromosomal abnormalities increases modestly from age 23 to 37 years, with a mean annual growth of 0.704% per year. Critically, at age 38 years, the incidence rate surged sharply by 14.79%, rising from 64.22% at age 37 years to 79.01% at age 38 years, and then increasing progressively to 94% by age 44 years (4). This distinct “surge” at age 38 years underscores a clear threshold beyond which the risk accelerates exponentially, confirming that women aged <38 years are within the pre-threshold portion of the age aneuploidy curve. This threshold pattern is further supported by large-scale PGT−A data. In a review of 15,169 consecutive trophectoderm biopsies, Franasiak et al. found that the rate of having no euploid embryo was lowest (2%–6%) in women aged 26–37 years. The rate increased to 33% by age 42 years, and to 53% by age 44 years (14). These findings confirm that women under 38 years of age experience a relatively stable, low baseline risk of aneuploidy, consistent with the modest effect size observed in our young cohort. Therefore, the lack of significance in this subgroup does not contradict the well-documented deleterious effect of aging on the oocyte; rather, it underscores that the clinical impact of maternal age is most robustly manifested once the critical threshold of 38 years is reached.

Notably, although the RPL group in this study was significantly older than the control group (33.0 ± 2.9 years vs. 31.2 ± 3.7 years), both the overall comparison and the age-stratified subgroup analyses revealed no significant differences in embryonic aneuploidy rates between the two groups. Although statistically significant, the absolute difference in mean age between the two groups was relatively small (approximately 1.8 years). The effect of maternal age on aneuploidy risk becomes more pronounced beyond the mid−30s, and both groups had mean ages below 35 years (4). The modest age gap may not have been sufficient to translate into a detectable difference in aneuploidy rates, especially given the inherent biological variability and the limited sample size in certain subgroups (15). Building upon the preceding argument, while the approximately 1.8−year age difference between the two groups was not sufficient to produce a statistically detectable disparity in embryonic aneuploidy rates, the same chronological gap did translate into significant differences in ovarian reserve parameters, including AFC and subsequent oocyte yield (16). This discrepancy can be attributed to the fact that the age-related decline in ovarian reserve follows a relatively linear and consistent trajectory, whereas the relationship between age and embryonic aneuploidy is more complex and exhibits a non-linear, exponential acceleration after the mid-30s (14).

In this study, the RPL group was approximately 2 years older than the control group and exhibited tendencies toward diminished ovarian reserve, as evidenced by lower AFC, fewer retrieved oocytes, and reduced E_2_ levels on the trigger day; however, their embryonic aneuploidy rates did not differ significantly from those of the control group. Therefore, in the management of patients with RPL aged <38 years, clinical decision-making should prioritize etiologies beyond embryonic chromosomal abnormalities. Routine application of PGT-A may offer limited value in this specific population and should be undertaken only after careful patient counseling.

## Data Availability

The original contributions presented in the study are included in the article/supplementary material. Further inquiries can be directed to the corresponding author.
